# Skewed distribution of proinflammatory CD4^+^CD28^null ^T cells in rheumatoid arthritis

**DOI:** 10.1186/ar2286

**Published:** 2007-09-07

**Authors:** Andreas ER Fasth, Omri Snir, Anna AT Johansson, Birgitta Nordmark, Afsar Rahbar, Erik af Klint, Niklas K Björkström, Ann-Kristin Ulfgren, Ronald F van Vollenhoven, Vivianne Malmström, Christina Trollmo

**Affiliations:** 1 Rheumatology Unit, Department of Medicine, Karolinska Institutet, Karolinska University Hospital, Stockholm, Sweden; 2 Department of Medicine, Center for Molecular Medicine, Karolinska Institutet, Karolinska University Hospital, Stockholm, Sweden; 3 Center for Infectious Medicine, Department of Medicine, Karolinska Institutet, Karolinska University Hospital Huddinge, Stockholm, Sweden

## Abstract

Expanded populations of CD4^+ ^T cells lacking the co-stimulatory molecule CD28 (CD4^+^CD28^null ^T cells) have been reported in several inflammatory disorders. In rheumatoid arthritis, increased frequencies of CD4^+^CD28^null ^T cells in peripheral blood have previously been associated with extra-articular manifestations and human cytomegalovirus (HCMV) infection, but their presence in and contribution to joint manifestations is not clear. In the present article we investigated the distribution of CD4^+^CD28^null ^T cells in the synovial membrane, synovial fluid and peripheral blood of RA patients, and analysed the association with erosive disease and anti-citrullinated protein antibodies. CD4^+^CD28^null ^T cells were infrequent in the synovial membrane and synovial fluid, despite significant frequencies in the circulation. Strikingly, the dominant TCR-Vβ subsets of CD4^+^CD28^null ^T cells in peripheral blood were often absent in synovial fluid. CD4^+^CD28^null ^T cells in blood and synovial fluid showed specificity for HCMV antigens, and their presence was clearly associated with HCMV seropositivity but not with anti-citrullinated protein antibodies in the serum or synovial fluid, nor with erosive disease. Together these data imply a primary role for CD4^+^CD28^null ^T cells in manifestations elsewhere than in the joints of patients with HCMV-seropositive rheumatoid arthritis.

## Introduction

T cells are likely to play an important role in the pathogenesis of rheumatoid arthritis (RA) (reviewed in [1]). In the synovial joint, infiltrating T cells are predominantly of the CD4^+ ^phenotype and are often found in the proximity of B cells and macrophages. These T cells could either represent cells potentiating the function of infiltrating leukocytes or represent suppressive regulatory T cells. Neither specific autoantigens nor autoreactive T cells have so far been conclusively demonstrated in RA. However, a distinct population of oligoclonally expanded proinflammatory CD4^+ ^T cells is found with increased frequencies in peripheral blood in RA patients compared with healthy control individuals [2-4]. These cells display a proinflammatory phenotype, are terminally differentiated, express a variety of NK cell-related receptors and lack the co-stimulatory molecule CD28; the cells are therefore often referred to as CD4^+^CD28^null ^T cells [5,6].

The presence of these CD4^+^CD28^null ^T cells in peripheral blood has been associated with human cytomegalovirus (HCMV) seropositivity, extra-articular manifestations and cardiovascular disease in RA patients [7-9]. Despite increased frequencies of CD4^+^CD28^null ^T cells in the circulation of RA patients, however, their contribution to erosive disease is still unclear: while studies from Pawlik and colleagues and Goronzy and colleagues found associations between circulating CD4^+^CD28^null ^T cells and erosive disease [4,10], Martens and colleagues and Gerli and colleagues did not observe such associations [3,9].

We had a unique opportunity to investigate the presence of these CD4^+^CD28^null ^T cells in the synovial membrane, the synovial fluid and peripheral blood from the same patients in a large cohort of RA patients. The association with erosive disease and the levels of antibodies to citrullinated peptides/antigens was examined. Furthermore, CD4^+^CD28^null ^T cells isolated from the synovial fluid were investigated with regard to antigen specificity and selective recruitment to the joint.

## Materials and methods

### Patients

One hundred and twenty-eight patients with RA were enrolled in the study. All fulfilled the American College of Rheumatology criteria for RA and attended the Rheumatology Clinic at Karolinska University Hospital, Stockholm, Sweden for corticosteroid injections of inflamed joints [11]. Before the corticosteroid injections, synovial fluids were acquired from the knee joints (*n *= 128), the elbow (*n *= 1) or the shoulder joints (*n *= 2). Eighty per cent of the patients were women, median age of 56 years (range, 25–82 years) and a median disease duration of 9 years (range, 0–45 years).

Assessment of erosive disease was performed by radiographic evaluations of the ankle joints or wrist joints by the same two rheumatologists. Radiographic changes in one or more joints were found in 51 out of 70 (73%) patients included in these analyses. The majority of the patients were treated either with nonsteroidal anti-inflammatory drugs, with systemic or local corticosteroid treatment, with methotrexate alone or in combination with corticosteroids (prednisolone), or with TNF blockers alone or in combination with methotrexate. Some patients were untreated.

This study was approved in compliance with the Helsinki Declaration by the Ethics Committee of the Karolinska University Hospital, and all patients and healthy subjects gave informed consent.

### Arthroscopy and synovial biopsies

Knee joint synovial biopsies were acquired according to a previously described procedure [12]. Biopsies were taken at the site of inflammation, either close to cartilage or not close to cartilage, defined as either less than 1.5 cm or more than 1.5 cm from cartilage, respectively.

### Three-colour immunofluorescence microscopy

Frozen unfixed synovial biopsy sections were fixed with acetone. Sections were incubated overnight with the cocktail of primary antibodies – CD244 (R&D Systems, Minneapolis, MN, USA), CD4 (Becton Dickinson, San Jose, CA, USA), CD3 (DakoCytomation, Glostrup, Denmark) – or the isotype control antibodies – goat IgG (Caltag Laboratories, Burlingame, CA, USA), mouse IgG_1 _(DakoCytomation) and rabbit immunoglobulin (DakoCytomation). Excess of antibodies were washed away before incubation with the secondary antibodies – anti-sheep/goat immunoglobulin-biotin (The Bidning Site, Birmingham, UK), avidin-Oregon Green 488 (Molecular Probes, Eugene, OR, USA), anti-mouse IgG-Rhodamine RedTM-X (Jackson ImmunoResearch Laboratories, West Grove, PA, USA) and anti-rabbit IgG-AMCA (Jackson ImmunoResearch).

Stained tissue sections were examined with a Leica DM RXA2 microscope (Leica Microsystems, Wetzlar, Germany) equipped with a Leica DC 300F (Leica Microsystems DI, Cambridge, UK) digital colour video camera connected to a PC computer. Photographs were analysed with Leica IM500 software (Leica Microsystems, Heerbrugg, Switzerland).

CD4^+^CD28^null ^T cells were identified by morphologically cell-like structures with co-localized immunostainings of CD3, CD4 and CD244, and were manually quantified in independent analyses performed by two persons. CD4^dim ^macrophages/monocytes and NK cells [13], which also might express CD244, could be excluded using the combination of CD3 and CD4 in the three-colour stainings to identify CD4^+ ^T cells. The density of CD4^+^CD28^null ^T cells were calculated by dividing the number of CD4^+^CD28^null ^T cells by the total area of infiltrating T cells, measured with Image J software version 1.34s (National Institutes of Health, Bethesda, MD, USA).

### Flow cytometry

The frequency of CD4^+^CD28^null ^T cells in peripheral blood and synovial fluid was analysed by four-colour flow cytometry (FACSCalibur instrument; Becton Dickinson Immunocytometry Systems, San Jose, CA, USA) in peripheral blood mononuclear cells and synovial fluid mononuclear cells after Ficoll separation (Ficoll-Paque Plus; GE Healthcare Biosciences AB, Uppsala, Sweden). The antibodies used were CD3-FITC, CD28-APC (Pharmingen; Becton Dickinson, San Diego, CA, USA), CD4-PerCP (Becton Dickinson, San Jose) and CD244-PE (Immunotech, Marseille, France).

The TCR-Vβ usage was determined by the IOTest1 Beta Mark kit (Beckman Coulter, Marseille, France). The TCR-Vβ stainings were combined with antibodies to CD4 and CD28 (see above) to identify CD4^+^CD28^null ^T cells and CD4^+^CD28^+ ^T cells.

Peripheral blood mononuclear cells and synovial fluid mononuclear cells stimulated with HCMV antigens (see below) were analysed by flow cytometry after immunostaining with IFNγ-FITC, CD28-PE, CD3-APC and CD14-APC-Cy7 (all from Becton Dickinson, San Diego, CA, USA) and CD4-PerCp (Becton Dickinson, San Jose, CA, USA).

Flow cytometric data were analysed with CellQuest software (Becton Dickinson, Franklin Lakes, NJ, USA) or FlowJo software (Tree Star Inc., Ashland, OR, USA). The frequency of CD4^+^CD28^null ^T cells was calculated as the percentage of CD28-negative cells in the gated CD3^+^CD4^+ ^population.

### Functional assays

The functional capacity of CD4^+^CD28^null ^T cells from the synovial fluid and peripheral blood were assessed by IFN-γ production. Two million peripheral blood mononuclear cells and synovial fluid mononuclear cells from eight patients were either stimulated with plate-bound anti-CD3 antibodies (OKT-3) at 0 or 0.1 μg/ml for 4 hours, or by 2 μg/ml pp65 and immediate early HCMV antigens (JPT Peptide Technologies GmbH, Berlin, Germany) for 8 hours.

Activated cells were either detected by secretion (MACS Secretion Assay; Miltenyi Biotec, Bergisch Gladbach, Germany) or by upregulation of intracellularly stored IFN-γ (Becton Dickinson, San Diego, CA, USA) according to the manufacturers' protocols. The frequency of IFN-γ secreting CD3^+^CD4^+^CD28^null ^T cells was analysed by flow cytometry (see above).

### Enzyme-linked immunosorbent assay

The anti-CCP2 test (Immunoscan RA, Mark 2; Euro-Diagnostica, Arnhem, The Netherlands) was used to determine the levels of anti-citrullinated peptide/protein antibodies (ACPA) in the serum and synovial fluid. A cutoff value of 25 U/ml was used according to the manufacturer's instructions. Serum and synovial fluid samples were diluted equally (1:50) and were analysed on the same plate.

The presence of anti-HCMV IgG and IgM antibodies in the serum and the synovial fluid, from the same time point as the screening of CD4^+^CD28^null ^T cells, was tested in an enzygnost anti-HCMV/IgG ELISA and an enzygnost anti-HCMV/IgM ELISA (Dade Behring, Marburg, Germany). Sera from HCMV-seronegative patients were further examined for detection of IgG against HCMV using antigens prepared from a HCMV clinical isolate (C6) using an ELISA as previously described by Rahbar and colleagues [14]. Control antigen was prepared from uninfected fibroblasts.

### Statistical analyses

Comparisons of nonparametrically distributed data in two independent groups or compartments were performed by the Mann–Whitney test. The Spearman test for correlation was used for analyses of covariation of two nonparametrically distributed data.

## Results

### CD4^+^CD28^null ^T cells are scarce in the synovial membrane

**Figure 1:**
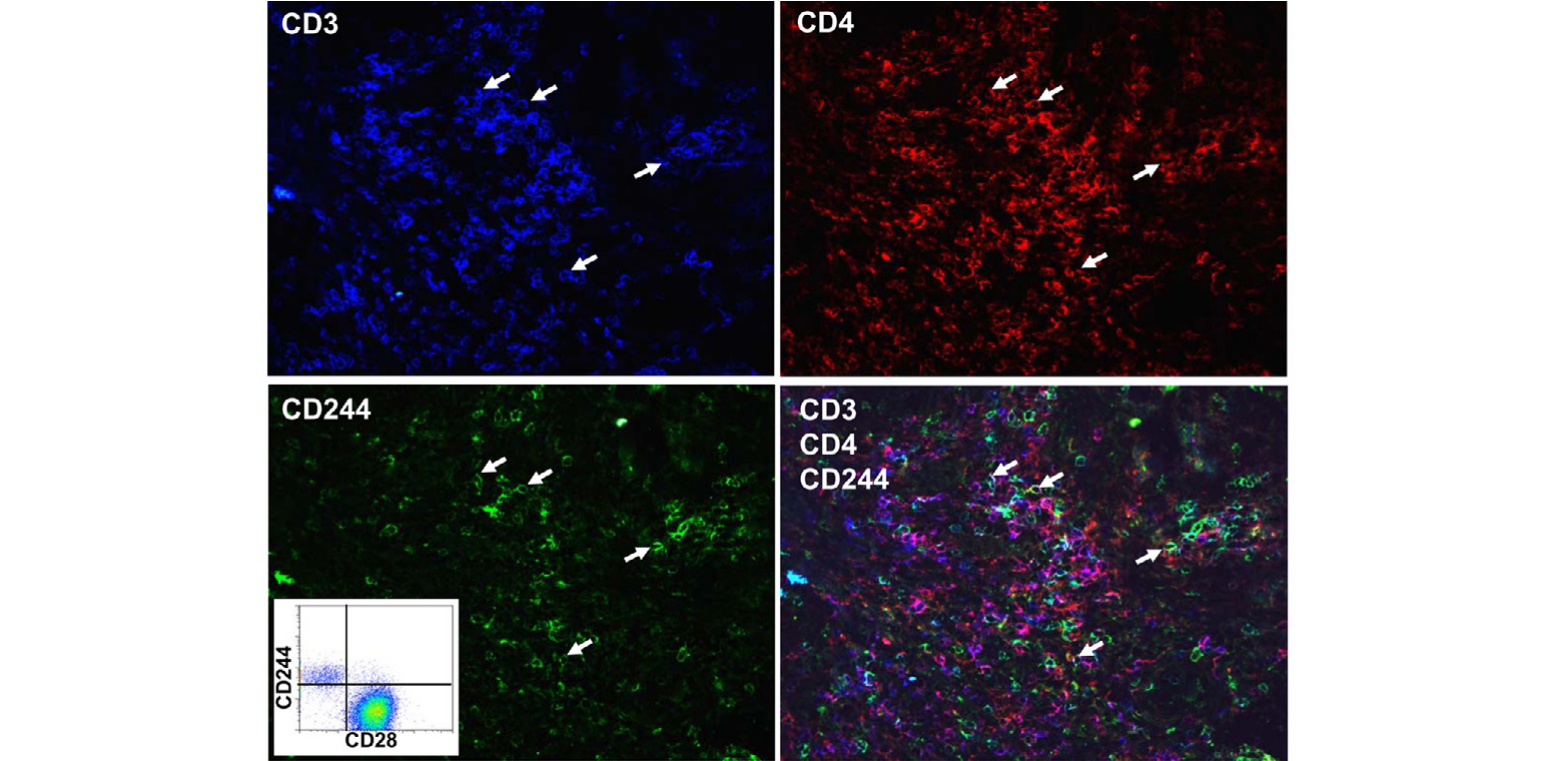
CD4^+^CD28^null ^T cells are rare in the synovial membrane. Representative immunostaining of one patient, using three-colour immunofluorescence microscopy, to identify CD4^+^CD28^null ^T cells in the inflamed synovial membrane. The four photographs depict each marker alone or superimposed of the same area of Patient 3 (Table 1): CD3 (blue), CD4 (red) and CD244 (green). Arrows indicate CD4^+^CD28^null ^(CD3^+^CD4^+^CD244^+^) T cells. Original magnification, ×32. Inserted flow cytometry panel displays the expression CD244 and CD28 on CD3^+^CD4^+ ^T cells in peripheral blood.

To investigate the presence of CD4^+^CD28^null ^T cells in the synovial membrane, we used three-colour immunofluorescence microscopy. This technique utilizes our findings of CD244 expression by CD4^+ ^T cells as a specific marker for the CD28^null ^subset in peripheral blood of patients with RA and muscle tissue of patients with myositis (Figure 1) (Fasth *et al*., submitted).

CD244 is hitherto mainly described as a receptor regulating activation of NK cells after interaction with its ligand CD48 [15]. CD244 might have similar functions on T cells, but this is not yet fully understood [16]. Instead of detecting T cells lacking CD28, CD4^+^CD28^null ^T cells in synovial membrane biopsies were identified by co-expression of CD244, CD3 and CD4 (Figure 1). Two patient groups were analysed: patients having less than 0.5% and patients having more than 7% of circulating CD4^+^CD28^null ^T cells (Table 1). In all 11 patients, as expected, CD3-positive cells were abundant in the synovial membrane, and a majority of the CD3-positive cells also expressed CD4 (Figure 1). Only small numbers of CD4^+^CD28^null^CD244^+ ^T cells were found. They were evenly distributed and without correlation with the size of the T-cell infiltrates, the frequencies of CD4^+^CD28^null ^T cells in peripheral blood or with ongoing medication. From these results we conclude that the vast majority of CD4^+^CD28^null ^T cells do not home specifically to the synovial membrane despite significant frequencies in the circulation.

**Table 1:** Summary of patients investigated for CD4^+^CD28^null ^T cells in synovial membrane biopsies

Patient	Gender, age (years)	Disease duration (years)	Treatment^a^	Erosive disease	CD3^+ ^in synovial membrane^b^	CD4^+^CD28^null ^T cells			
						
						Peripheral blood (%)	Synovial fluid (%)	*n*/T-cell infiltrate^c^	*n*/mm^2 ^T-cell infiltrate^c^
>5% CD4^+^CD28^null ^T cells in peripheral blood									
1	Female, 69	1	P, M	-	++	18	6	3	35
2	Female, 45	15	N, L	+	+	9.8	10.4	2.5	54
3	Female, 67	2.75	N	+	++	7.6	6.5	5	47
4	Female, 51	33	N, P	+	+++	13	1.5	4.7	48
5	Male, 74	26	N, P, E	+	++	8.6	2.7	0.8	5
6	Male, 38	22	N, M, E	+	++	8	0.8	2	39
<1% CD4^+^CD28^null ^T cells in peripheral blood									
7	Male, 45	13	N	+	+	0	0.4	1.3	53
8	Male, 25	0.6	N, P, M	-	++	0	0.2	3	51
9	Female, 60	Several years	N, D	-	++	0	0.1	0	0
10	Male, 41	1	P	+	+++	0.4	0.8	4.5	67
11	Female, 66	10	M	+	++	0.4	0.6	0	0

^a^P, prednisolone; M, methotrexate; N, nonsteroidal anti-inflammatory drug; L, leflunomid; E, etanercept; D, depomedrol. ^b^Relative size of infiltrating CD3^+ ^cells in the synovial membrane specimen: +, <25% T cells in the tissue area; ++, approximately 50% T cells in the tissue area; +++, >75% T cells in the tissue area. ^c^Average numbers of CD4^+^CD28^null ^T cells in one to five analysed infiltrates.

### CD4^+^CD28^null ^T cells are restricted to HCMV-seropositive patients and are less frequent in synovial fluid than in peripheral blood

**Figure 2:**
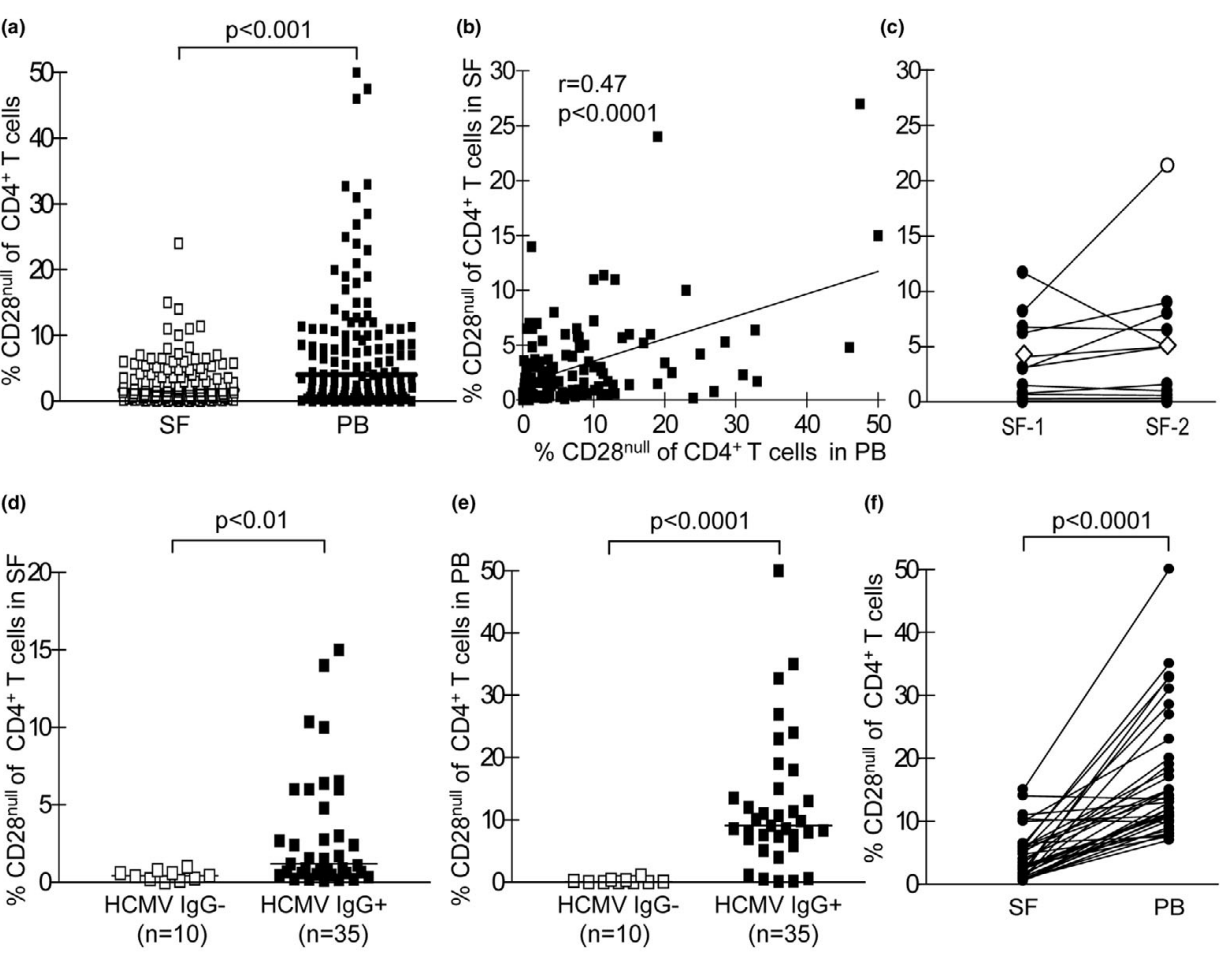
Skewed distribution of CD4^+^CD28^null ^T cells in synovial fluid and peripheral blood. **(a) **Paired samples of synovial fluid (SF) and peripheral blood (PB) from 128 rheumatoid arthritis patients were compared for the frequency of CD4^+^CD28^null ^T cells by flow cytometry. **(b) **Frequencies of CD4^+^CD28^null ^T cells in SF tend to be higher in patients with large populations in PB. **(c) **Comparison of the frequency of CD4^+^CD28^null ^T cells in SF from two different synovial compartments. Open circle, elbow; open squares, shoulder joints. Frequencies of CD4^+^CD28^null ^T cells in **(d) **SF and **(e) **PB in patients seronegative and seropositive for human cytomegalovirus (HCMV). **(f) **Frequencies of CD4^+^CD28^null ^T cells in paired PB and SF of patients seropositive for HCMV.

We then analysed the presence of CD4^+^CD28^null ^T cells in the synovial fluid by screening synovial fluid samples from 128 patients by flow cytometry. CD4^+^CD28^null ^T cells could be detected in synovial fluid at a median frequency of 1.9% (range 0–27%). These percentages, however, were significantly lower than in paired peripheral blood samples (median, 4.4%; range, 0–50%; *P *< 0.001) (Figure 2a). Despite significant differences in the two compartments, patients with the largest population of CD4^+^CD28^null ^T cells in the synovial fluid still tended to have the highest frequencies in peripheral blood (*r *= 0.47, *P *< 0.0001) (Figure 2b).

Analysis of 14 patients with two simultaneous synovial fluid samples demonstrated that the presence of CD4^+^CD28^null ^T cells in one inflamed joint reflects the occurrence also in other affected joints (Figure 2c). Patient groups undergoing different medical treatments (untreated, nonsteroidal anti-inflammatory drug, methotrexate or TNF blockade) did not have different frequencies of CD4^+^CD28^null ^T cells in the peripheral blood or the synovial fluid (data not shown).

Increased frequencies of circulating CD4^+^CD28^null ^T cells in patients with RA have been associated with HCMV infection; whether this is also valid for synovial CD4^+^CD28^null ^T cells is not known. Strikingly, we found significant CD4^+^CD28^null ^T-cell populations only in the synovial fluid of HCMV-seropositive individuals (*P *< 0.01) (Figure 2d). Comparative analysis of the frequencies of CD4^+^CD28^null ^T cells in the synovial fluid and peripheral blood of HCMV IgG-seropositive patients clearly showed that CD4^+^CD28^null ^T cells were less frequent in synovial fluid compared with peripheral blood also in this subset of patients (*P *< 0.0001) (Figure 2f). The serum titres of anti-HCMV IgG correlated with the levels in the synovial fluid (*r *= 0.87, *P *< 0.0001; data not shown). Seven patients (15%) displayed both anti-HCMV IgM and anti-HCMV IgG antibodies, indicating a recent or ongoing infection, but the frequency of CD4^+^CD28^null ^T cells in the circulation and synovial fluid was similar to IgG-seropositive patients lacking IgM (data not shown).

These data demonstrate that CD4^+^CD28^null ^T cells are only present in the synovial fluid and peripheral blood of RA patients seropositive for HCMV, and demonstrate that, despite significant frequencies in peripheral blood, CD4^+^CD28^null ^T cells are infrequent in the synovial compartment.

### CD4^+^CD28^null ^T cells from synovial fluid are functional and reactive to HCMV antigens

**Figure 3:**
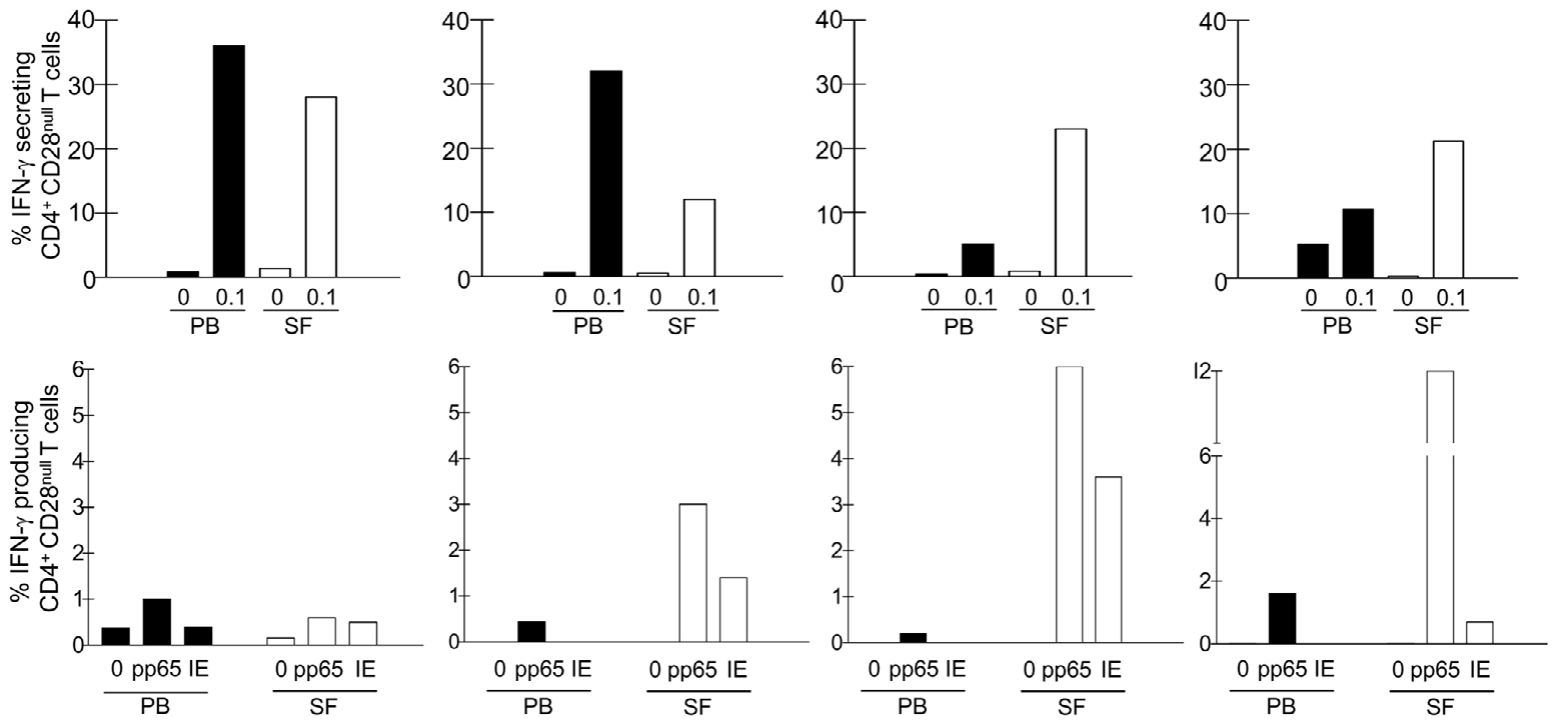
CD4^+^CD28^null ^T cells from peripheral blood and synovial fluid show human cytomegalovirus specificity. **(upper panel) **The functional capacity of CD4^+^CD28^null ^T cells in peripheral blood (PB) and synovial fluid (SF) was investigated after stimulation of mononuclear cells from paired PB and SF from rheumatoid arthritis patients by plate-bound anti-CD3 antibodies. **(lower panel) **The reactivity of CD4^+^CD28^null ^T cells in paired PB and SF to human cytomegalovirus (HCMV) antigens was analysed after stimulation by the pp65 or immediate early (IE) antigens.

We have previously reported that CD4^+^CD28^null ^T cells from the peripheral blood rapidly respond to low TCR stimulation mimicked by a low concentration of anti-CD3 antibodies [6]. To investigate whether CD4^+^CD28^null ^T cells in the synovial fluid displayed the same hyperresponsiveness, we measured the frequency of IFN-γ-secreting CD4^+^CD28^null ^T cells in mononuclear cells from synovial fluid. Indeed, these cells showed a similar response as CD4^+^CD28^null ^T cells from the peripheral blood (Figure 3, upper panel), indicating that CD4^+^CD28^null ^T cells in the synovial compartment have a capacity to function as local effector T cells.

With the strong association between CD4^+^CD28^null ^T cells and HCMV seropositivity we also investigated whether IFN-γ production could be provoked from CD4^+^CD28^null ^T cells in peripheral blood and synovial fluid, after stimulation by the HCMV-derived pp65 and immediate early antigens. Interestingly, synovial fluid-derived CD4^+^CD28^null ^T cells from all investigated patients showed specificity for these antigens (Figure 3, lower panel). These results indicate that HCMV-derived peptides can activate at least a subset of CD4^+^CD28^null ^T cells to function as effector T cells.

### Selected subsets of CD4^+^CD28^null ^T cells have access to the inflamed joint

Hitherto we have shown that CD4^+^CD28^null ^T cells are less frequent in the synovial fluid compared with peripheral blood. We next investigated whether there was a selective recruitment of certain subsets of CD4^+^CD28^null ^T cells to the joint. Using antibodies detecting 24 different TCR-Vβ chains, we could identify TCR-Vβ subsets of the CD4^+^CD28^null ^T-cell population in both peripheral blood and synovial fluid. Two distinct patterns of TCR-Vβ distribution were found in the joint.

**Figure 4:**
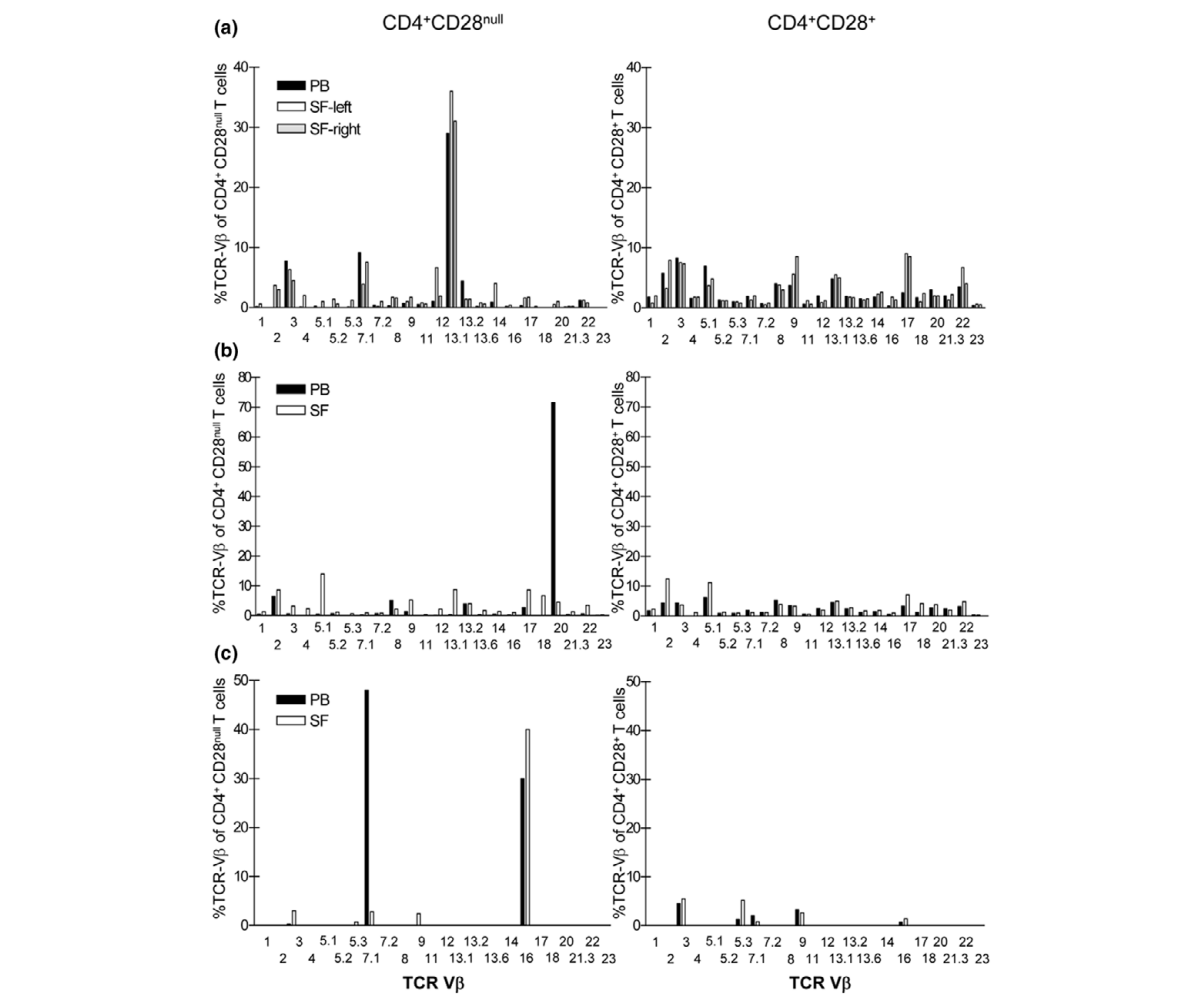
Restricted access of CD4^+^CD28^null ^T-cells subset to synovial fluid. **(a) **TCR-Vβ repertoire of CD4^+^CD28^null ^T cells (left) and CD4^+^CD28^+ ^T cells (right) from peripheral blood (PB, filled bars) and synovial fluid (SF, open bars) from one patient with similar frequencies of TCR-Vβ in the CD4^+^CD28^null ^populations in PB and SF. **(b) **TCR-Vβ repertoire of CD4^+^CD28^null ^T cells (left) and CD4^+^CD28^+ ^T cells (right) from PB and SF from one patient with different frequencies in TCR-Vβ in the CD4^+^CD28^null ^population in PB and SF. **(c) **TCR-Vβ repertoire of CD4^+^CD28^null ^T cells (left) and CD4^+^CD28^+ ^T cells (right) from PB and SF in one patient: one of the two dominant CD4^+^CD28^null ^T-cell TCR-Vβ subsets in PB was present in SF with even higher frequencies than in PB, while the access of the other dominant subset in PB was significantly restricted. In this patient, the T cells were only screened for selected TCR-Vβ chains.

In six of the 13 patients analysed, the dominant TCR-Vβ within the CD4^+^CD28^null ^T-cell population in peripheral blood was also present in the joint. In one such representative patient, with total frequencies of 7%, 1% and 1.5% CD4^+^CD28^null ^T cells in the circulation, in the left knee synovial fluid and in the right knee synovial fluid respectively, 29–36% of the CD4^+^CD28^null ^T cells in all three compartments expressed TCR-Vβ13.1 (Figure 4a). In contrast, synovial fluid from five patients displayed a clear exclusion of the dominant CD4^+^CD28^null ^T cells from peripheral blood. In the most prominent case, a patient with total frequencies of 9% CD4^+^CD28^null ^T cells in peripheral blood and 4% in the synovial fluid, 71.5% of the CD4^+^CD28^null ^T cells in the circulation expressed Vβ20 but only 4.5% of the cells in the joint expressed Vβ20. In contrast, there was an enrichment of TCR-Vβ5.1-expressing CD4^+^CD28^null ^T cells in the joint (Figure 4b). Patients with the restricted pattern did not display lower overall frequencies of synovial fluid CD4^+^CD28^null ^T cells as compared with patients with the same TCR-Vβ dominance in peripheral blood and synovial fluid. Neither did different medical treatments co-segregate with either pattern of TCR-Vβ distribution (data not shown). Interestingly, in one patient displaying two TCR-Vβ subsets covering more than 75% of the circulating CD4^+^CD28^null ^population, one of the subsets had access to, and was even significantly enriched in, the synovial fluid, while the other dominant subset was clearly restricted (Figure 4c). Two patients did not display any of these distinct patterns of TCR-Vβ distribution.

In all 13 patients, the distribution of TCR-Vβ subsets in the conventional CD4^+^CD28^+ ^T-cell population was not dramatically different in the synovial fluid from that in peripheral blood, as illustrated in Figure 4a–c. These results suggest that the access of CD4^+^CD28^null ^T cells to the joint is restricted to certain TCR-Vβ subsets, and that RA patients can be divided into two distinct groups with regard to different access of CD4^+^CD28^null ^T-cell subsets to the joint.

### Associations between CD4^+^CD28^null ^T cells, ACPA and erosive disease

**Figure 5:**
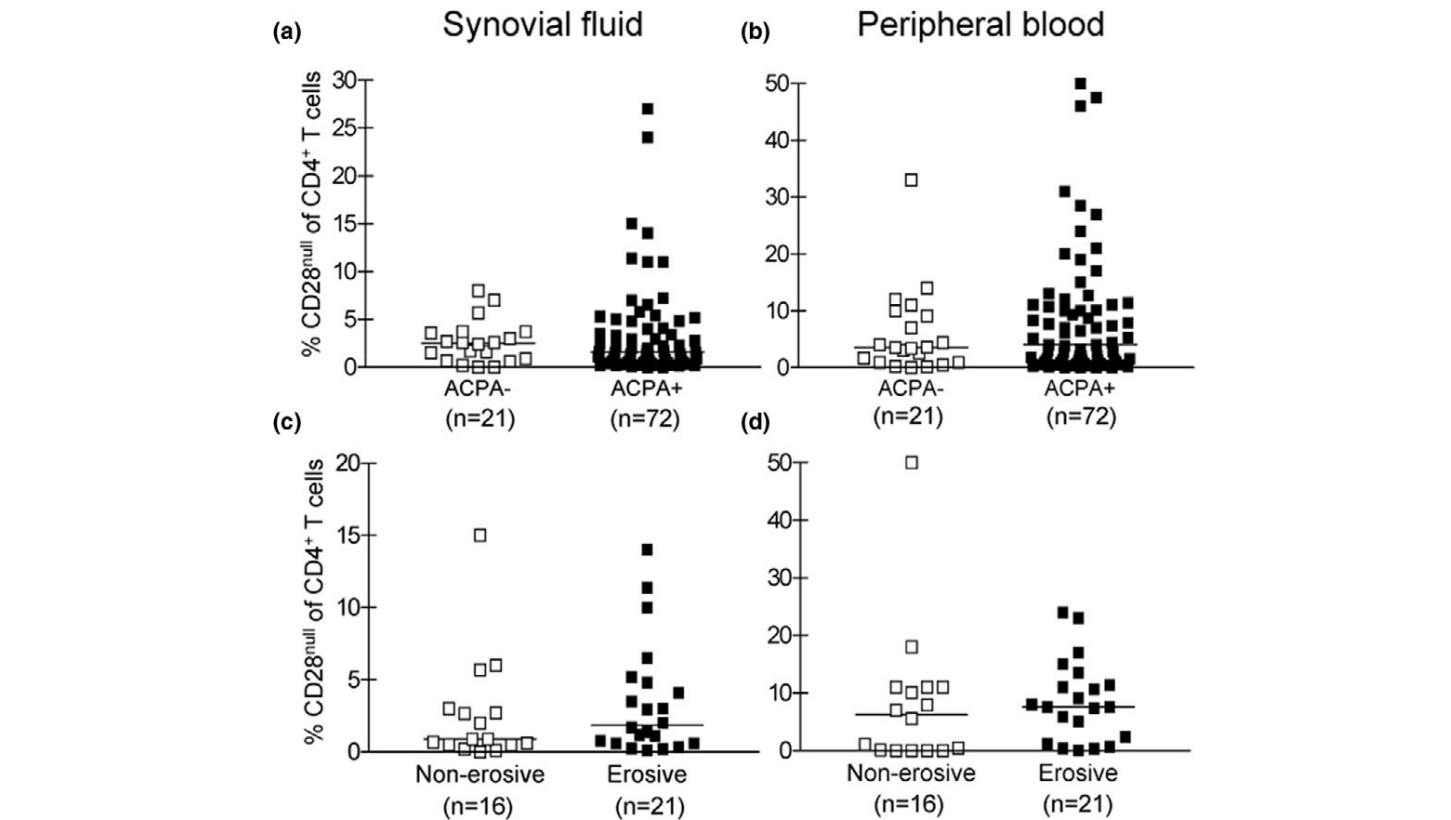
CD4^+^CD28^null ^T cells do not associate with erosive disease. Frequency of CD4^+^CD28^null ^T cells in paired samples of synovial fluid (left column) and peripheral blood (right column) from **(a), (b) **patients seronegative (ACPA^-^) and seropositive (ACPA^+^) for anti-citrullinated peptide/protein antibodies, and **(c), (d) **patients with or without erosive disease.

The presence of ACPA is RA specific and is an early predictor of an aggressive disease course [17,18]. We therefore analysed whether the frequencies of CD4^+^CD28^null ^T cells in the circulation and in the synovial fluid were associated with the presence of ACPA in these compartments. We found no differences in the frequencies of circulating or synovial CD4^+^CD28^null ^T cells in patients with or without ACPA in these locations (Figure 5a,b).

To investigate the association between CD4^+^CD28^null ^T cells and erosive disease, we allocated patients into two clearly distinguishable groups of either significant erosive disease (*n *= 21) or no signs of erosive disease (*n *= 16) according to radiographic evaluations. We had access to longitudinal follow-up data on patients without erosions up to 10 years after disease onset; therefore, patients allocated to the erosive group also had a disease duration of a maximum 10 years. Since subgroups of patients with different treatment did not display different frequencies of CD4^+^CD28^null ^T cells either in peripheral blood or in synovial fluid, treatment was not considered a parameter to stratify for in this analysis. The results indicated that CD4^+^CD28^null ^T cells were not specifically enriched in the joint or peripheral blood of patients with erosive disease (Figure 5c,d), which is in accordance with the lack of correlation with ACPA levels.

Increased frequencies of CD4^+^CD28^null ^T cells have been associated with extra-articular manifestations [3,4]. Three cases of such manifestations were reported in our cohort. One patient, rheumatoid factor-positive and HLA-B27-positive, with iritis had no CD4^+^CD28^null ^T cells either in blood or the synovial fluid. In contrast, two patients with pleurisy had 31% and 33% CD4^+^CD28^null ^T cells in peripheral blood and 2.3% and 6.4% in the synovial fluid, respectively. Despite only two patients, these observations are in line with the notion of an association between CD4^+^CD28^null ^T cells and extra-articular manifestations in RA.

Taken together, our data from peripheral blood and the inflamed synovial joint show no association between the presence of CD4^+^CD28^null ^T cells and joint destruction.

## Discussion

Herein we demonstrate that only minor populations of CD4^+^CD28^null ^T cells were present in the inflamed joints of RA patients, despite significant percentages in peripheral blood. The presence of CD4^+^CD28^null ^T cells in peripheral blood and the synovial fluid was strongly associated with HCMV IgG seropositivity, but not with ACPA or erosive disease.

Our results on the presence of CD4^+^CD28^null ^T cells in the synovial fluid were based on screening for CD3^+^CD4^+^CD28^- ^cells. It was therefore important to consider the stability of the CD28-negative phenotype. Data from *in vitro *experiments indicate that cytokines such as TNF and IL-12 in synovial fluid can modify CD28 expression, complicating the analyses of CD4^+^CD28^null ^T cells in the joint [19,20]. We believe our data, however, not to be biased by the cytokines present in the synovial fluid since there was no over-representation of synovial CD4^+^CD28^+ ^T cells expressing the TCR-Vβ chains preferentially expressed by peripheral blood CD4^+^CD28^null ^T cells (Figure 4). Interestingly, previous studies have shown a reduction in the frequency of CD4^+^CD28^null ^T cells in peripheral blood after TNF blockade [9,21-23]. In our cohort, neither TNF blockade nor any of the other most frequently used medical treatments (untreated, nonsteroidal anti-inflammatory drug, methotrexate) was associated with the distribution of CD4^+^CD28^null ^T cells in the synovial fluid, peripheral blood and synovial tissue. It is therefore likely that the effect of TNF blockade on CD4^+^CD28^null ^T cells is primarily seen when comparing the same patients before and after treatment, rather than comparing heterogeneous groups of patients with and without this treatment.

CD4^+^CD28^null ^T cells from both peripheral blood and synovial fluid demonstrated reactivity to HCMV-derived antigens. That the frequency of responding CD4^+^CD28^null ^T cells was higher in the synovial fluid compared with the peripheral blood does not necessarily mirror an accumulation of HCMV-reactive CD4^+^CD28^null ^T cells in this compartment, since the same effect was seen for CD4^+^CD28^+ ^T cells (data not shown). These differences might instead be due to a different status of accessory cells from the two compartments. We also analysed the HCMV specificity of the dominant TCR-Vβ subsets of CD4^+^CD28^null ^T cells from two patients comprising 20% and 29% of the total CD4^+^CD28^null ^population in both peripheral blood and synovial fluid. Interestingly, the TCR-Vβ dominant CD4^+^CD28^null ^T-cell subsets did not respond either to pp65 or to immediate early antigens, indicating that the TCR-Vβ dominant subsets might be reactive to antigens other than those considered immunodominant for HCMV (data not shown). This might be explained by a hypothesis suggested by Davenport and colleagues, who demonstrate that during chronic Esptein–Barr virus infections T-cell clones reactive to the most dominant epitopes rapidly decrease after primary infection and that clonotypes reactive to less dominant epitopes control the recurrent infections [24]. At present, however, we can not exclude that some of the CD4^+^CD28^null ^T cells with access to the joint have specificity for cartilage-derived and/or citrullinated candidate antigens.

It is intriguing that only certain subsets of CD4^+^CD28^null ^T cells reach the synovial fluid. Since we were not able to detect all possible TCR-Vβ chains, we cannot exclude that there is an even more pronounced discrimination of CD4^+^CD28^null ^T cells expressing nondetectable TCR-Vβ chains. The reason for this skewed distribution of CD4^+^CD28^null ^T cells in peripheral blood and synovial fluid, both with regard to subsets of CD4^+^CD28^null ^T cells and to the size of whole populations, can with present knowledge only be speculated upon. Owing to the strong association of CD4^+^CD28^null ^T cells to HCMV seropositivity, it is tempting to assume that the location or status of the HCMV infection plays an important role. Because of the increased frequencies in peripheral blood and exclusion from the synovial fluid, it is probable that tissues other than the rheumatic joint are the primary homing sites for CD4^+^CD28^null ^T cells in these patients. The few CD4^+^CD28^null ^T cells found in the joint could instead be a consequence of general patrolling initiated by infection in other tissues. A widespread distribution of virus-specific T cells in a site other than the actual site of virus infection has previously been demonstrated in mouse models [25]. Further investigations considering the expression of chemokine receptors/integrins, antigen specificity, location of the HCMV infection and the presentation of HCMV antigens are needed to clarify this issue. The frequency of CD4^+^CD28^null ^T cells in the synovial fluid does not necessarily reflect the situation in the inflamed synovia, although in our cohort the low CD4^+^CD28^null ^T-cell frequencies in the synovial fluid were in agreement with the modest numbers in the inflamed synovial membrane.

CD4^+^CD28^null ^T cells isolated from the synovial fluid could function as effector T cells by rapid secretion of IFN-γ. Interestingly, IFN-γ is only scarcely found in the T-cell infiltrates of the rheumatic synovial membrane and has therefore not been considered a key cytokine in the pathogenesis of RA [26]. Several reports instead indicate the importance of IL-17, and recent publications have further shown that IFN-γ counteracts the differentiation of IL-17-producing T cells [27-29]. Since CD4^+^CD28^null ^T cells produce IFN-γ but not IL-17 [6,30], it is possible that CD4^+^CD28^null ^T cells, if activated in the joint by secretion of IFN-γ, might even inhibit the synovial inflammation in RA.

The limited presence of CD4^+^CD28^null ^T cells in the synovial fluid, despite increased frequencies in peripheral blood and their equal distribution in patients with and patients without erosive disease, indicates no significant role for CD4^+^CD28^null ^T cells in the local inflammation driving joint destruction. Instead, these data indirectly support the previously suggested role for these cells in extra-articular manifestations and cardiovascular disease. That is, CD4^+^CD28^null ^T cells are exclusively present in HCMV IgG-seropositive RA patients and are reactive to HCMV antigens (present study and [7]), CD4^+^CD28^null ^T cells only have limited access to the inflamed joint despite increased frequencies in the circulation (present study), increased frequencies of CD4^+^CD28^null ^T cells do not correlate with erosive disease (present study and [3,9]), RA patients with high frequencies of circulating CD4^+^CD28^null ^T cells display increased risk for cardiovascular events compared with patients lacking CD4^+^CD28^null ^T cells [9,31], CD4^+^CD28^null ^T cells have been found in atherosclerotic plaques and can mediate lysis of endothelial cells *in vitro *[32,33], HCMV is frequently found in atherosclerotic and nonatherosclerotic vascular walls [34], and HCMV increases the thrombogenicity of endothelial cells [35].

CD4^+^CD28^null ^T cells and HCMV might not be the only mediators of cardiovascular events in RA, but these studies together strongly link CD4^+^CD28^null ^T cells and HCMV infection to cardiovascular events, which is found with increased prevalence and is the major cause of death in patients with RA.

## Conclusion

In the present study we have shown that CD4^+^CD28^null ^T cells in RA only are present in HCMV-seropositive patients and display a skewed distribution in the inflamed joint compared with that in peripheral blood. Consistent with the limited number of CD4^+^CD28^null ^T cells in the joints, these cells were not associated with joint destruction indicated by radiographic analyses or ACPA antibodies predictive of erosive disease.

## Abbreviations

ACPA = anti-citrullinated protein antibodies; ELISA = enzyme-linked immunosorbent assay; HCMV = human cytomegalovirus; IFN = interferon; IL = interleukin; RA = rheumatoid arthritis; TCR = T-cell receptor; TNF = tumour necrosis factor.

## Competing interests

The authors declare that they have no competing interests.

## Authors' contributions

AERF was responsible for the study design, performed laboratory work (preparation of blood and synovial fluid samples, flow cytometry analyses, development, analyses of the immunofluorescence microscopy, and HCMV reactivity assays), statistical analyses, interpretation of the data, and drafted the manuscript. OS performed and interpreted data from *in vitro *stimulation assays as well as ACPA ELISAs of serum and synovial fluid. AATJ performed and analysed the immunofluorescence stainings. BN contributed to the clinical evaluation of the patient cohort together with EaK, who also performed the arthroscopies. AR carried out and evaluated the results from the HCMV ELISAs. NKB set up the flow cytometric assays for investigation of the CD244 expression. A-KU was responsible for the biobank of arthroscopic biopsies and participated in the development of immunofluorescence stainings. RvV contributed to the clinical evaluations of patients and manuscript preparation. VM and CT were the principle investigators and participated equally in the planning and coordination of the study, interpretation of data, and drafting the manuscript. All authors read and approved the final manuscript.
